# Supplementary material for: Using the Avocado to Test the Satiety Effects of a Fat-Fiber Combination in Place of Carbohydrate Energy in a Breakfast Meal in Overweight and Obese Men and Women: A Randomized Clinical Trial
Source: Nutrients. 2019 Apr 26;11(5):952. doi: 10.3390/nu11050952 (PMC6567160; doi:10.3390/nu11050952)
Supplement: Supplementary file 1 [file nutrients-11-00952-s001.pdf]

Supplement Table S1. Hass avocado nutrition facts.

	Hass avocado, Whole	Hass avocado, Half
Weight (g)	136	68
Calories (kcal)	217.6	108.8
Fat Calories (kcal)	179.4	89.7
Saturated fat Calories (kcal)	26.0	13.0
Protein (g)	2.7	1.4
Carbohydrate (g)	11.6	5.8
Fiber (g)	9.1	4.6
Sugar (g)	0.9	0.5
Fat (g)	19.9	10
Saturated Fat (g)	2.9	1.5
Monounsaturated Fat (g)	13.3	6.7
Polyunsaturated Fat (g)	2.5	1.2

Source: USDA Nutrient database (https://ndb.nal.usda.gov/ndb/foods/show/09038?fgcd=&manu=&format=&count=&max=25&offset=&sort=default&order=asc&qlookup=Hass+Avocados&ds=&qt=&qp=&qa=&qn=&q=&ing=, accessed April 22, 2019)
